# Reviews of Dental Literature

**Published:** 1905-10

**Authors:** 


### Reviews of Dental Literature.

The Antimicrobic Action of Iodine.[1] By Guy C. Kinnaman, M.D., Assistant Instructor in Skin and Venereal Diseases, Chicago Policlinic, Chicago.

PREFACE.

The uniform satisfaction given by the iodine catgut prepared after the method of Claudius,[1] which has been used by Dr. Nicholas Senn in his clinical work, led him to employ a solution of iodine for irrigation in infected wounds and also for hand sterilization. The results obtained demonstrated, clinically, the marked potency of iodine in solution as an antiseptic. Its continued use for several years has shown it to be far superior to any antiseptic previously employed.

In order to determine the absolute value of this solution, a series of experiments have been carried out on a number of different micro-organisms *in vitro.* A sufficient number of different organisms and a great number of tubes have been used. Therefore one is justified in assuming that the conclusions arrived at from a careful study of the series of experiments represent as nearly as possible the position of iodine in the family of antiseptics. When we try to make an accurate comparison between the members of this numerous family, however, we find we cannot do so because of the widely different methods of procedure employed by the different investigators. In addition, the majority of the methods show one or more points of possible error. Not for the purpose of comparison, but with the idea in view of presenting the experimental findings of various men on the more important antiseptic substances, there has been incorporated in this research a short bibliography of antiseptics in general.

*Bichloride Mercury.*—Kyle[2] found that it took $^1/_{1000}$ solution bichloride ten hours to insure no growth of tetanus bacillus. Koch[3] determined that a $^1/_{20000}$ solution killed anthrax spores in ten minutes. Klein,[4] how-

---

[1] Work done as Fellow in Surgery at the Senn Laboratory, Rush Medical College. An abstract prepared from the original article in the Journal of the American Medical Association, with consent of the publisher.

ever, found that anthrax spores were not influenced by a $^1/_{100000}$ solution acting for one hour. Evans,[5] from experiments on *Bacillus anthracis* in liquid media, reported that a $^1/_{15000}$ for one mniute and a $^1/_{20000}$ for thirty minutes were effective. In regard to the spores he found that a $^1/_{1000}$ for fifteen minutes and $^1/_{1500}$ for one hour were effective. Likewise, a $^1/_{1000}$ for five minutes gave growth. On *Staphylococcus pyogenes aureus,* the same author states that a $^1/_{200}$ solution for fifteen minutes killed, while a $^1/_{250}$ solution for one hour impaired vitality. Tarnier and Vignal,[6] using thread method of Koch, found that a $^1/_{1000}$ solution for two minutes killed both *Streptococcus* and *Staphylococcus pyogenes aureus.* Harrington,[7] employing the same method, states that where the threads are moist an exposure of over fifteen minutes to a $^1/_{1000}$ solution is necessary to kill the *Bacillus pyocyaneus,* while if threads are dry five seconds are sufficient. On *Staphylococcus aureus,* either on moist or dry threads, from five to ten minutes kills. On *Bacillus anthracis,* either dry or moist threads, an exposure to a $^1/_{1000}$ solution for thirty minutes has no bactericidal effect. Hence, he states that bichloride of mercury is a greatly overrated disinfectant. Burgess,[8] who also used the thread method in his experiments on *Bacillus coli communis,* found that a $^1/_{750}$ solution for one minute was effective, as were a $^1/_{2000}$ solution for five minutes and a $^1/_{7500}$ for ten minutes. Sternberg,[9] from experiments on gonococcus, states that 0.005 per cent. bichloride solution for two hours kills. He concludes that in bichloride we have a germicidal agent of the highest value.

*Carbolic Acid.*—Kyle[2] found that the tetanus bacillus had to be exposed to a five per cent. carbolic acid solution for six hours in order to assure no growth. Cabbot,[10] on adding five per cent. carbolic acid to a putrefactive solution and then transferring several drops of mixture to an aseptic medium, discovered that after five seconds' exposure no putrefaction occurred in fresh tubes. Evans[5] found that on anthrax bacillus a $^1/_{100}$ solution for five minutes, a $^1/_{150}$ solution for fifteen minutes, and a $^1/_{175}$ solution for thirty minutes, were effective. On the spores, he got a growth after four hours' exposure to a five per cent. solution. A twenty-four hours' exposure, however, was sufficient to insure no growth. A $^1/_{40}$ solution on *Staphylococcus aureus* for fifteen minutes gave no growth. Burgess,[8] in his work on *Bacillus coli communis,* states that a ten per cent. solution kills in one minute, a 2.5 per cent. in five minutes, a one per cent. in thirty minutes, and a 0.75 per cent. in one hour. Blyth[11] found that a ten per cent. solution for four hours had no effect on anthrax spores. A twenty-four-hour exposure, however, proved efficient. He used the glass-rod method. On the gonococcus and micrococcus of abscess pus, Sternberg[9] found that the action of an 0.8 per cent. solution for two hours killed the organisms.

*Alcohol.*—From experiments on the germicidal effect of alcohol, Harrington[12] concludes: "Alcohol of any strength has absolutely no effect on anthrax bacillus; sixty to seventy per cent. alcohol is the most efficient strength against non-spore-forming bacteria, *i.e., Staphylococcus pyogenes aureus* and *albus,* typhoid bacillus, bacillus of diphtheria, being usually effective in five minutes."

Salzwedel and Elsner,[13] using thread method, state that fifty-five per cent. (by weight alcohol) was most destructive to staphylococcus pus, requiring in fresh pus from five to six minutes to be effective. They state that this solution is not quite equal to a $^1/_{1000}$ solution of bichloride, but is as efficient as a three per cent. carbolic solution. Sternberg[9] found that with a two-hour exposure, a ninety-five per cent. alcohol was fatal to gonococci, while a forty per cent. solution did the work on the micrococcus of abscess pus.

*Hydrogen Peroxide.*—In his work on the tetanus bacillus, Kyle[2] states that after a forty-eight-hour exposure to hydrogen peroxide the bacillus still grew. On the other hand, Gibier[14] finds, from experiments he has made with a 3.2 per cent. hydrogen peroxide solution in water on *Bacillus anthracis, Bacillus typhosus, Streptococcus pyogenes,* etc., that after some minutes of contact (exact time not stated) the bacteria could not be cultivated from bouillon, hence were destroyed.

*Formaldehyde.*—Shaw[15] determines that a $^1/_{2000}$ solution of formaldehyde broth prevented the growth of *Bacillus pyocyaneus.* Pottevin[16] finds that five centigrams of formaldehyde to a litre of bouillon stops growth of *Bacillus diphtheriæ, pyocyaneus, typhosus* and the *Staphylococcus pyogenes aureus.* Burgess[8] concludes that on *Bacillus coli communis,* a $^1/_{30}$ solution is destructive in one minute, a $^1/_{50}$ solution in five minutes, and a $^1/_{150}$ solution in thirty minutes.

*Lysol.*—Burgess,[8] who included lysol in his experiments on *Bacillus coli,* found that a $^1/_{10}$ aqueous solution killed it in one minute, a $^1/_{30}$ solution in five minutes, a $^1/_{250}$ solution in thirty minutes, and a $^1/_{300}$ solution in one hour.

*Boric Acid.*—Burgess[8] also found that a saturated solution of boric acid was entirely wanting in antiseptic powers, no matter how long the exposure. Sternberg[9] came to a like conclusion from his work on gonococcus and micrococcus of abscess pus.

*Phenic Acid.*—On streptococci and staphylococci Tarnier and Vignal[6] state that a three per cent. aqueous solution of phenic acid is efficient in seventeen minutes, a two per cent. solution in eighteen minutes. Chamberland,[17] from his experiments, finds that anthrax bacilli are killed by a forty-eight-hour exposure to a $^1/_{400}$ solution of phenic acid.

*Salicylic Acid.*—A $^1/_{240}$ solution of salicylic acid for three minutes kills putrefactive organisms, according to Cabbot.[10]

*Methyl Violet.*—Stillings,[18] as his findings from experiments, states that methyl violet is three times as strong as bichloride in its action on anthrax bacillus and quite as effective as it is on *Staphylococcus pyogenes aureus.*

*Nicotine.*—Herbert,[19] reviewing the work of Pécholier (1883) on the action of nicotine in alcoholic solution on bacteria; that of Vincent Tassinari de Pise (fumes of tobacco, in 1888); and that of Vissalli (fumes of tobacco, in 1888); and from added experiments of his own (nicotine in alcohol solution), says in conclusion that "nicotine does not appear gifted with very powerful antiseptic properties, for one-third of a drop (sufficient to kill a pigeon in a few seconds) does not prevent the development of certain bacteria in three cubic centimetres of bouillon." But he concludes from experiments of Tissinari on tubercle bacillus and spirillum of Asiatic cholera, and from his own on bacillus diphtheria that it attenuates the virulence of microbes. Junor[20] reports a case of tetanus cured by local application of infusion of tobacco.

*Mercuric Cyanide.*—Harrington,[21] using the thread method, found that a $^{1}/_{1000}$ solution of mercuric cyanide had to act in thirty minutes to kill *Staphylococcus pyogenes albus,* while a three-hour exposure on *S. aureus* showed no antiseptic effect. Hence it is impractical.

*Radium.*—It was found by Van Buren,[22] from his experiments on *Bacillus typhosus, Bacillus pyocyaneus* and *Staphylococcus pyogenes aureus,* that radium, acting from eight to thirteen hours, was negative in antiseptic action. Pfeiffer[23] also shows its negative action on *Bacillus typhosus* and the cholera spirillum.

*Collargol.*—Fortesque-Brickdale[24] states " in solution of 0.5 per cent. and under, collargol has no bactericidal effect on *Streptococcus pyogenes* or *Staphylococcus pyogenes aureus* after a twenty-four-hour exposure to a one-day-old culture. (Plate Method.)" A one per cent. solution gave no growth.

*Turpentine.*—Marx[25] found that it required $^{1}/_{500}$ turpentine acting for an hour to hinder the growth of anthrax. Forty minutes was required for *Staphylococcus pyogenes aureus.*

*Glycerin.*—Copeman,[26] from numerous experiments, fully covered by controls, on *Staphylococcus pyogenes aureus* and *albus, Streptococcus pyogenes, Bacillus anthracis, subtilis, coli communis, pyocyaneus, diphtheriæ,* and *tuberculosis* determines: " (1) No visible development of organisms employed takes place in presence of more than thirty per cent. glycerin in beef peptone broth; (2) all organisms, except coli and subtilis, are killed in less than one minute in the presence of thirty per cent. to forty per cent. glycerin in the cold."

*Potassium Permanganate.*—According to Cabbot,[10] a $^{1}/_{960}$ aqueous solution of permanganate potassium requires over six minutes to stop putrefaction in a putrefactive solution. Sternberg[9] found that 0.12 per cent. permanganate acting on gonococcus and micrococcus of abscess pus destroyed them.

### BIBLIOGRAPHY OF ANTISEPTICS CONTAINING IODINE IN CHEMICAL COMBINATION.

*Biniodide of Mercury.*—On the *Staphylococcus aureus* and the *Streptococcus,* Tarnier and Vignal[6] have shown that a 1/1000 solution of biniodide of mercury is destructive in nine minutes; a 1/2000 solution in twenty-two minutes. On *Bacillus coli communis,* Burgess[8] states that "biniodide of mercury is by far the strongest and best antiseptic (of the ones he tried), and it is not affected by albumin." In his table he places 1/1000 solution effective in one minute; 1/5000 in five minutes; 1/20000 in ten minutes; 1/80000 in thirty minutes; 1/200000 in one hour.

*Lithio-Mercuric Iodide.*—Rosenberger,[27] working with lithio-mercuric iodide (thirty-four per cent. mercury, sixty-five per cent. iodine, one per cent. lithium) and using thread method, found that on *Staphylococcus pyogenes aureus*, when threads were moist, 1/1000 solution for thirty seconds killed; 1/5000 for one minute did the same. When threads were dry, 1/1000 for one minute killed; likewise 1/5000 for three minutes. On *Streptococcus pyogenes* (dry threads) 1/1000 for thirty seconds killed; 1/5000 for thirty seconds killed; threads moist, 1/1000 for thirty seconds was effective; 1/5000 required two minutes. On anthrax bacillus 1/1000 solution was necessary for fifteen minutes when threads were dry, and for thirty minutes when moist threads were used. He concludes that this is a much more powerful germicide than the usual mercuric salts.

*Iodoform.*—The experimental results differ greatly from the clinical. A great deal of experimental work has been done on iodoform. Its action has been tested on various organisms by numerous investigators, and their findings seem to indicate that it possesses no bactericidal properties of any moment. The men who have so determined are Heyn and Rovsing,[28] Riedlin,[29] Baumgarten,[30] Kronacher,[31] Buchner,[32] Kunz,[33] Schnirer,[34] Lubbert,[35] and Tilanus.[36] On the other hand, the work of Mikulicz,[37] who, on adding iodoform to putrefying liquids, saw putrefaction sometimes stopped and sometimes to continue; that of Maylard,[38] who states that it retards but does not stop the development of anthrax bacillus; and that of Stohegoleff,[39] who found that tubercle bacilli inoculated on five per cent. iodoform bouillon were dead in forty-eight hours, seems to indicate that under certain conditions (*i.e.*, where free iodine is eliminated) it has a slight antiseptic value.

Clinically, when the iodoform comes in contact with tissues, more free iodine is eliminated, hence the results are much better than experiments would indicate. Its use in tubercular joint injections needs no comment. Vinke[40] reports favorably in two cases of anthrax dressed with iodoform. Sormani[41] found by wounding rabbits and guinea-pigs and injecting them with tetanus bacilli and then treating them with different antiseptics that iodoform was the most active of all, and if applied at once tetanus did not appear. Pryor[42] reports thirty-seven cases of puerperal sepsis with but four deaths treated by packing uterus and widely opened cul-de-sac with iodoform gauze, free iodine being liberated and shown to be present in the urine.

*Iodine.*—The antiseptic action of iodine was discovered by Davaine [43] in 1873. He found that a 1/12000 solution caused the virulence of anthrax to disappear in thirty minutes. Tarnier and Vignal,[6] in their work on *Staphylococcus pyogenes aureus* and *Streptococcus pyogenes,* found that a 1/1000 solution of potassium iodide required over an hour to kill, a 1/500 solution required fifteen minutes, and a 1/300 solution required eight minutes. White [44] states that he has made a number of tests and observed that "in strength of 1/1000 iodine solution in bouillon no growth of streptococcus occurs. *Staphylococcus aureus* will not grow in strength of 1/5000." Davaine [45] determines that a 1/170000 solution of iodine in contact with anthrax blood for fifty to sixty minutes renders it harmless to inject into guinea-pigs. Sternberg [9] states that a 0.2 per cent. solution destroys gonococcus acting for two hours. Claudius,[1] in his article describing his method of sterilizing catgut by immersing in a solution of one part iodine, one part potassium iodide, and water one hundred parts, for eight days and then transferring to three per cent. carbolic acid to rinse off excess of liquid iodine, remarks that from his experiments he has found that a one per cent. solution kills *Staphylococcus aureus* in three minutes and anthrax in twenty hours. He states further that such a long contact is unnecessary (1/5000 solution kills anthrax spores in a short time).

Clinically, iodine solutions have been used with excellent results in treating anthrax. Sereins [46] reported two cases of anthrax cured by injecting a 1/100 solution around and into pustule. Brochin [47] has also reported two cases successfully treated by the same method. Human actinomycosis has been cured by the daily injection of two cubic centimetres of a ten per cent. iodine solution, as shown by Gaucher.[48]

In concluding the bibliography, it is only necessary to call attention to the great number of different substances employed as antiseptics to show that, as yet, the ideal germicide is a negative quantity.

### TECHNIC OF EXPERIMENTAL RESEARCH.

*Solution Employed.*—A solution of iodine in sodium iodide and water was prepared after the following formula:

| | |
|---|---|
| Iodine | 2.5 gms. |
| Sodium iodide | 5.5 gms. |
| Aqua (sterilized) | 250.0 cc. |

This gave a 1/100 solution. From this solution, by aqueous dilution, strengths of 1/200, 1/300, 1/500, and 1/1000 were made. In preparing the mother solution, accuracy was observed in weighing the iodine and sodium iodide. The same care was followed in making dilutions. The glass containers were sterilized.

*Asepticity of Solutions.*—The varying strengths of solutions were now tested to see whether or not they were, of themselves, sterile. For this purpose the plate method was employed. Three drops of solution were used to inoculate a melted agar tube from which a plate was made. From each strength solution three plates were made. After incubating six days every plate was free from colonies, hence sterile. To confirm results inoculations from the different strengths were made on six per cent. glycerin potato and incubated six days. The result was again negative in character.

METHOD OF CONDUCTING EXPERIMENTS.

In conducting the several series of experiments, an effort was made to employ a method which would offer the minimum possibility of error, and, at the same time, approach, as nearly as possible, to the exact relation of bacteria to the body in disease. In other words, the media employed were solid in every case, and the growths on them were allowed to become marked, both in extent and thickness, before beginning experiment. Thus, by the thickness of the growth, the penetrating power of the liquid could be ascertained.

The micro-organisms on which the action of the iodine solutions was tried were various enough to permit of definite deductions being made from the results on the antimicrobic action of iodine. These micro-organisms were: (1) *Bacillus prodigiosus;* (2) *Bacillus anthracis* and spores; (3) *Bacillus tuberculosis;* (4) *Staphylococcus pyogenes aureus;* (5) *Streptococcus pyogenes;* (6) *Blastomyces dermatitis;* (7) *Actinomyces bovis.* On these various growths in the test-tubes, the iodine solutions were deposited by means of a sterilized pipette until the surface of the growth was completely covered. After varying periods of time, which are designated in the tables, small loops of growth, thus acted on, were transferred to tubes of sterile media and well inoculated, and then put in incubator and watched for growth. No effort was made to wash the inherent iodine solution from the loops because of the danger of washing away the bacteria. For each time of exposure, four tubes were used, each one being covered by a control tube. It is well to note that the bacterial growths transferred were macroscopic and not microscopic in size.

SUMMARY OF EVIDENCE ON ANTHRAX.

Considering first the strength of solution and time of exposure to same necessary to produce the death of *Bacillus anthracis* plus spores, the evidence shows that a 1/200 solution for one hour or a 1/100 solution for ten minutes procures death. An exposure to a 1/100 solution for less than ten minutes gives a growth. The second consideration or the inhibitory effect, depending as it does on the two factors,—*i.e.*, time of exposure and strength of solution,—first shows itself after an exposure of the organism to a 1/1000 solution for sixty minutes. The inhibitory effect is marked. The effect is also shown by an exposure of fifteen minutes to a 1/500 solution, but to a lesser degree. As the time of exposure is increased, the inhibitory effect becomes more and more marked. Likewise, as the strength of solution employed is increased, the inhibitory action becomes progressively more powerful. If, after the death point is reached, we decrease the time of exposure, we again come into the sphere of diminishing inhibition.

SUMMARY OF EVIDENCE ON PRODIGIOSUS.

In making a summary of the evidence, there are two factors to be considered: (1) The strength of solution and time of exposure to same necessary to produce the death of the micro-organism; (2) the inhibitory action of solutions. Considering (1), therefore, we find that a solution less than one per cent., acting for an hour, does not destroy *Bacillus prodigiosus.* To cause the death of this organism it is necessary for a 1/100 solution of iodine to act for ten minutes. A shorter period of exposure,—*i.e.*, nine, eight, and five minutes,—gives a growth. On considering (2) it is readily seen that two items have to do with the inhibitory effect. These are, strength of solution and period of exposure. Thus, a solution acting for a certain period of time shows no inhibitory effect, but if the time of exposure is increased, such an effect appears. Conversely, as the strength of the solution is increased, the time of exposure necessary to produce an inhibitory effect becomes less. Bearing these two facts in mind, we find that in this series a slight inhibitory effect is first shown by a 1/1000 solution after an hour's exposure. A 1/500 solution begins to show this effect after thirty minutes' exposure; a 1/300 solution after fifteen minutes. Thus, progressively, as the time of exposure to each strength of solution is lengthened, and the strength of the solution itself is increased, the inhibitory action becomes more marked until the death point of the germ is reached. And, if we try to decrease the time of exposure necessary to produce death, we reach the inhibitory stage again.

### SUMMARY OF EVIDENCE ON TUBERCULOSIS.

The evidence shows that to produce the death of the *Bacillus tuberculosis* an exposure to a 1/200 iodine solution for one hour or to a 1/100 solution for seven minutes is necessary. An exposure of less than seven minutes results in a growth. When we come to the consideration of the inhibitory effect we find that a 1/1000 solution for one hour is without effect. Inhibition begins to be shown slightly by a 1/500 solution for fifteen minutes. From this point on, as the period of exposure is increased and as the solution employed becomes of greater strength, the inhibitory effect becomes progressively more and more marked until the death point is reached. Then, if we try to lessen the time of exposure to the necessary strength, we come into the sphere of inhibition which decreases as the exposure decreases.

### BIBLIOGRAPHY.

1. Claudius: Deutsche Zeitschrift für Chirurgie, 1902, vol. lxiv. pp. 489–494.

2. Kyle: Therapeutic Gazette, 1892, vol. viii. p. 163.

3. Koch: Mittheilungen aus dem k. Gesundheitsamte, vol. i. 1881.

4. Klein: Local Gov. Bd. Rep., London, 1885–86.

5. Evans: Guy's Hospital Report, London, 1890, 3 s., vol. xxxii. pp. 195–252.

6. Tarnier and Vignal: Arch. de méd. expér. et d'anat. path., Paris, 1890, vol. ii. p. 477.

7. Harrington: Boston Medical and Surgical Journal, 1903, vol. cxlviii. pp. 445–439.

8. Burgess: Lancet, London, 1900, vol. i. p. 1798.

9. Sternberg: American Journal of Medical Science, 1883, vol. lxxxv. pp. 321–343.

10. Cabbot: Boston Medical and Surgical Journal, 1879, vol. ci. p. 75.
11. Blyth: Proc. Royal Society, Edinburgh, 1884, vol. xii. p. 638.
12. Harrington: Boston Medical and Surgical Journal, 1903, vol. cxlviii. p. 551.
13. Salzwedel and Elsner: Berliner klin. Wochenschr., 1900, vol. xxxvii. pp. 496–500.
14. Gibier: Verhandl. d. X, International Medical Congress, 1890, Berlin, 1891, v. 15 Abth., pp. 124, 125.
15. Shaw: Journal Hygiene, London, 1903, vol. iii. p. 162.
16. Pottevin: Annales de l'Institut Pasteur, 1894, p. 804.
17. Chamberland: Acad. de Science, Paris, 1883, vol. xcvi. p. 1088.
18. Stillings: Lancet, London, 1891, i. p. 872.
19. Herbert: L'art méd., Paris, 1896, vol. lxxxiii. pp. 176–193.
20. Junor: Edinburgh Medical Journal, 1866–67, vol. xii. p. 684.
21. Harrington: Boston Medical and Surgical Journal, 1904, vol. cl. p. 43.
22. Van Buren: American Medicine, 1903, vol. vi. p. 1021.
23. Pfeiffer: Berliner klinische Wochenschrift, 1903, vol. xl. p. 641.
24. Fortescue-Brickdale: Bristol Medico-Chirurgical Journal, 1903, vol. xxi. p. 343.
25. Marx: Centralbl. f. Bakteriol., Jena, 1902, vol. xxxiii. p. 75.
26. Copeman: Rep. Brit. Assn. Adv. Sc., London, 1896, p. 987.
27. Rosenberger: American Medicine, 1904, vol. vii. p. 1021.
28. Heyn and Rovsing: Fortschritte der Medicin, 1887, Bd. V. pp. 33–47.
29. Riedlin: Arch. f. Hyg. München U. Leip., 1887, vol. vii. p. 310.
30. Baumgarten: Berliner klinische Wochenschrift, 1887, vol. xxiv. p. 354.
31. Kronacher: Münchener med. Wochenschrift, 1887, vol. xxxiv. p. 545.
32. Buchner: Münchener med. Wochenschrift, 1887, vol. xxxiv. p. 465.
33. Kunz: Arbeiten a. d. Pathologische Institut zu Königsberg, 1887.
34. Schnirer: Wiener medicin. Presse, 1887, Nos. 36–38.
35. Lubbert: Fortschritte d. Medicin, Bd. V, 1887, pp. 330–345.
36. Tilanus: Münchener medicinische Wochenschrift, vol. xxxiv. p. 309.
37. Mikulicz: Langenbeck's Archiv, vol. xxvii. pp. 196–239.
38. Maylard: Annals of Surgery, 1890, vol. xi. pp. 17–33.
39. Stohegoleff: Arch. de méd. expér. et. d'Anat. Path., 1894, vol. vi. pp. 813–837.
40. Vinke: St. Louis Courier of Medicine, vol. xiii. p. 125.
41. Sormani: Riforma Medica, 1890, January, pp. 11–13.
42. Pryor: New York Medical Journal, 1903, vol. lxxviii. p. 356.
43. Davaine: Dictionaire Encyclopédique des Sciences méd., p. 335.
44. White: Amer. Jour. of Obstetrics, 1904, vol. xlix. p. 605.
45. Davaine: Bull. Acad. de méd., 1880, 2 S., vol. ix. p. 763.
46. Sereins: Union méd., Paris, 1887, 3 S., vol. xliv. p. 470.
47. Brochin: Gaz. d. hôp., Paris, 1880, vol. liii. p. 1010.
48. Gaucher: Ann. de Derm. et Syph., 1903, 4 S., vol. iv. p. 837.

An Interesting Case in Practice.[1] By J. H. Carter, L.D.S.I., D.D.S. (Phil.).

The following case, which first came under my notice in 1899, has been treated off and on by myself and partner up to the present time, puzzling and worrying us both very much.

Mr. ——, a gentleman about fifty years of age, residing near Leeds, of fair complexion and healthy appearance, called, desiring me to scale and polish his teeth; also to remove any sharp edges I could find in connection with them. This I promptly did. I should explain that his mouth appeared normal and his natural dentures better than the average. In a little time he came again, complaining of roughness behind the lower incisors. These were carefully re-examined and some further scaling done to remove any possible fraction of tartar that might have been overlooked on the previous occasion; also I thoroughly polished the lingual surfaces again. To my surprise, in a week or two he again called, with the same complaint as before, especially in connection with the two central incisors. I did not quite know what to do, being so satisfied this roughness did not exist, but as he was so persistent, I repeated the polishing process and polished as I have never polished before, particularly their approximal surfaces. Each time on going away he expressed satisfaction and said they were better.

In a month or so, on my way to the station one day, a gentleman driving a carriage stopped me; well, it was my patient, who said he was no better, and would I see him again. When he called, he remarked that he was more convinced than ever that there was a roughness; that this bothered him extremely, his attention being perpetually drawn to it by the annoyance it caused. He compared it to the feeling as if the tip of his tongue was rubbing against sand-paper; indeed, he had been to his doctor, who could find nothing wrong, and who referred him back again to me, as it was quite a dental case. My patient suggested the advisability of having the two centre teeth extracted; to this, of course, I demurred. Wondering if there was anything wrong with his tongue, I examined this organ, but it appeared quite healthy and normal. Thinking the trouble might be partly mental or imaginary,

---

[1] Read before the Leeds and District Section of the North Midland Branch, March 14, 1905.

and feeling that doing something was the best way of getting the thing off his mind, I did still further polishing. Time went on, and again he hailed me from his carriage, stating that he was no better and what was to be done? In my despair I suggested his calling again, an appointment was made, but, not being able to find anything wrong when he came, I told him to divert his mind from his teeth; that his tongue must be morbidly sensitive, and that he had got into a way of fidgeting about it. In order to thoroughly polish the sides of these teeth, as they were set very closely together, I got my patient to wear some tape between them for the purpose of separating, so that I could use a polishing disk or other means. At the next visit he cheerfully informed me that the presence of the tape had nearly cured him. This gave me the idea that if only all the interstices were filled up I should succeed, so, putting on rubber dam, I packed each space with gutta-percha and made all very smooth. To my great annoyance in a little time he called, saying this material had become loose and come away. I saw there was no good in renewing it, for the little movement in each tooth had destroyed the adhesion, and I was sure nothing would hold. Feeling that my patient might be losing confidence in me and perhaps go elsewhere, and thinking a change of dentists might be of advantage, also being heartily weary of the matter, I suggested that my partner should take him in hand. He, knowing the history of the case, adopted very heroic treatment, passing files and disks between the incisors, rounding off everything in all directions, and polishing to such an extent that even the cutting edges were made round like knobs. As all this treatment failed as before, my partner recommended the wearing of a gold shield or plate, covering the lingual surfaces of all the six front teeth and affixing by clasps to the bicuspids; this arrangement afforded some relief, and it was worn for a considerable period.

About this time I was again accosted in the street by my patient, who complained that he was not comfortable and did not like wearing the plate; also he did not know why I had transferred him to some one else, and desired me to take him in hand again. Feeling that this plate was likely to cause damage, a happy inspiration came to me. I suggested discarding it and crowning, or rather encasing, the two offending teeth with gold. This I did, and I am glad to say matters were improved a good deal; yet the case is not altogether satisfactory. I should explain that one of the lateral incisors, not being crowned, is sensitive where the enamel has been polished away; this tooth is now much shorter than its neighbors, and appears to be getting much abraded. I saw the patient quite recently, and was much discouraged on being told that his thoughts were still constantly recurring to his teeth. Almost from the beginning my diagnosis was that either something was wrong with the tongue or the evil was imaginary; if the former, then it must be due to exaggerated or enlarged papillæ, or abnormal sensitiveness. This troublesome case got at last on my nerves, and I was in dread, when on my way to the station, lest I should again have my friend stopping me to say that he was no better, and what was to be done?

I have recently been in correspondence with his doctor regarding him, and he writes: "I am of the opinion that, for the last two or three years he has suffered from neurasthenia, connected with or due to business anxieties. During this time he has had the great responsibility of a failing business, in which not only his own fortune, but those of his brother and sisters, have been involved. Two or three years ago he complained much of right brachial neuralgia, after this had troubled him for some months, he suffered from left intercostal neuralgia and wandering pains about the neck and sternum, making himself and his doctor anxious as to the possibility of angina; finally, a spasmodic condition of the tongue muscles, with complaint of the sharp edges of his teeth, revealed the neurasthenic neurosis. I must say that I for a long time attributed all these things to irregular gout, as there was often an accompanying lithæmia." One authority says of neurasthenia, it is "a peculiar condition of the nervous system, a deviation more or less from the normal state and characterized by a loss of resistance, the latter in its turn producing an increased irritability and debility." Another says it is "a morbid irritability, an irritable weakness, an exaggerated sensibility. All kinds of unpleasant sensations and even vivid pains are of usual occurrence in neurasthenic individuals. The development of neurasthenia is specially favored by overwork, more particularly of a mental kind, by late hours, disappointment, grief, and care. It is of a subjective nature, and even in the most painstaking examination no objective foundation can be found, so that it is generally regarded as imagination or exaggeration; it causes the patient, however, enough trouble and discomfort to make him lose his happiness for a considerable period of his life."

In conclusion, this case may serve to suggest that we occasionally have mysterious and inexplicable symptoms presenting themselves, without sufficient or assignable cause; cases which we are unable to diagnose satisfactorily, and which perhaps belong more to the domain of the medical man than the dental surgeon.—*British Dental Surgeon.*

